# Use of an orthovoltage X-ray treatment unit as a radiation research system in a small-animal cancer model

**DOI:** 10.1186/1756-9966-27-57

**Published:** 2008-10-28

**Authors:** Luis-Alberto Medina, Blanca-Ivone Herrera-Penilla, Mario-Alberto Castro-Morales, Patricia García-López, Rafael Jurado, Enrique Pérez-Cárdenas, José Chanona-Vilchis, María-Ester Brandan

**Affiliations:** 1 Instituto de Física, Universidad Nacional Autónoma de México (UNAM), México D.F. 04510, Mexico; 2 División de Investigación Básica, Instituto Nacional de Cancerología (INCan), San Fernando 22, México D.F., 14080, Mexico; 3 Unidad de Investigación Biomédica en Cáncer INCan-UNAM, San Fernando 22, México D.F, 14080, Mexico; 4 Departamento de Patología, Instituto Nacional de Cancerología. San Fernando 22, 14080, México D.F, 14080, Mexico

## Abstract

**Background:**

We explore the use of a clinical orthovoltage X-ray treatment unit as a small-animal radiation therapy system in a tumoral model of cervical cancer.

**Methods:**

Nude mice were subcutaneously inoculated with 5 × 10^6 ^HeLa cells in both lower limbs. When tumor volume approximated 200 mm^3 ^treatment was initiated. Animals received four 2 mg/kg intraperitoneal cycles (1/week) of cisplatin and/or 6.25 mg/kg of gemcitabine, concomitant with radiotherapy. Tumors were exposed to 2.5 Gy/day nominal surface doses (20 days) of 150 kV X-rays. Lead collimators with circular apertures (0.5 to 1.5 cm diameter) were manufactured and mounted on the applicator cone to restrict the X-ray beam onto tumors. X-ray penetration and conformality were evaluated by measuring dose at the surface and behind the tumor lobe by using HS GafChromic film. Relative changes in tumor volume (RTV) and a clonogenic assay were used to evaluate the therapeutic response of the tumor, and relative weight loss was used to assess toxicity of the treatments.

**Results:**

No measurable dose was delivered outside of the collimator apertures. The analysis suggests that dose inhomogeneities in the tumor reach up to ± 11.5% around the mean tumor dose value, which was estimated as 2.2 Gy/day. Evaluation of the RTV showed a significant reduction of the tumor volume as consequence of the chemoradiotherapy treatment; results also show that toxicity was well tolerated by the animals.

**Conclusion:**

Results and procedures described in the present work have shown the usefulness and convenience of the orthovoltage X-ray system for animal model radiotherapy protocols.

## Background

Small animal models of human cancer have been used around the world to develop and evaluate several treatments schemes in cancer research [1]. Radiation treatments have been applied in different animal tumor models to evaluate and validate potential treatments in humans [2-4]. However, the absence of a dedicated small-animal irradiator, explicit for radiation treatments, has led to the use of clinical devices such as ^60^Co irradiators, linacs, brachytherapy sources, etc., that normally do not conform doses to a target volume in small animals, and also are cumbersome to manipulate in experiments. In addition, systemic secondary effects related with nonconformal irradiations, such as immune response, bone marrow depletion, etc., may mask the effect of interest in tumor, or in the evaluation of chemotherapy agents combined with radiotherapy.

Recently, research groups have been working in the development of dedicated small-animal irradiation systems that potentially will delivery conformal doses to specifically chosen targets, either tumors or normal tissues [5-8]. However, these systems are still in the process of prototype design or in validation studies, and their availability and cost will possibly limit the access to this technology. This might be particularly important in small research centers or developing countries, not able to afford the acquisition of a small animal irradiator immediately.

In this report, we explore the potential use of a clinical orthovoltage X-ray treatment unit as a small-animal radiation system. These kinds of units are available in several hospitals and medical centers, and their easy manipulation and accessibility, as compared with linacs and ^60^Co units, could turn them into an important tool for radiation therapy experiments with small animals.

To demonstrate the usefulness of this system in radiation therapy experiments, in the present work, a tumor model of cervical cancer was developed in nude mice and treated with concurrent chemoradiotherapy based on cisplatin. Our group is interested in the evaluation of chemoradiotherapy protocols for cervical cancer based on cisplatin, gemcitabine and different radiosensitizers. Cisplatin has been considered the most active cytotoxic agent for treatment of squamous carcinoma of the cervix, and is commonly used in the clinical practice in combination with radiation treatments [9-11]. Gemcitabine has shown very potent radiosensitizing properties in cervical cancer and is widely used in concurrent chemoradiotherapy with cisplatin [9,12]. Simultaneous administration of chemotherapy and radiotherapy which act upon different phases of the cell cycle may provide synergestic tumor response [13,14], but there are still questions with respect to this hypothesis. For this reason, preclinical evaluations of different treatments schemes based on chemoradiotherapy procedures need to be performed in tumor animal models. The results and methodology described in this work have shown the feasibility for the use of an orthovoltage X-ray system in chemoradiotherapy research protocols in tumor models.

## Methods

### Orthovoltage X-ray system specifications

**Figure 1:**
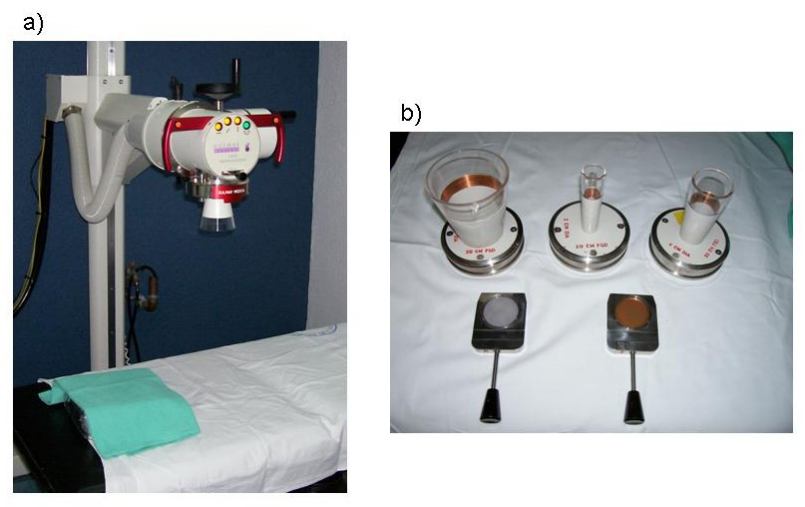
a) Orthovoltage X-ray unit (Gulmay D3225); b) filters and applicator cones.

The orthovoltage X-ray treatment unit used in this experiment (D3225, Gulmay Medical Ltd., UK) (Figure 1) uses a metal ceramic X-ray tube capable of delivering X-rays at voltages from 20 to 220 kV. The unit has several applicator cones (with different length and openings) that define treatment distances and field sizes. The anatomical dimensions in the present study have led us to choose 150 kV, 10 mA, added filtration of 2.25 mm Al and 0.15 mm Cu, and a treatment distance (source-to-surface distance, SSD) equal to 20 cm and a 2 cm diameter circular field size. Under these conditions the effective energy of the X-ray beam was measured to be E_eff _= 60.9 keV (Half Value Layer, HVL = 0.47 mm Cu) [15,16] and the absorbed dose rate to water at SSD was 2.16 Gy/min. Dose rate measurements were performed by the Medical Physics personnel from the Radiotherapy Department at Instituto Nacional de Cancerología (INCan) using a calibrated end-window parallel-plate ionization chamber (Marcus Advance, PTW, Germany).

### Tumor model

**Figure 2:**
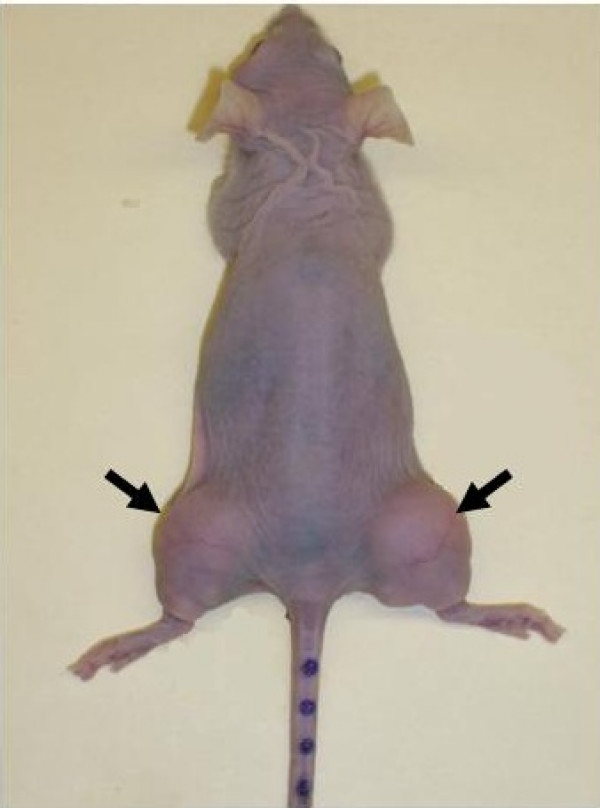
**Tumor xenograft of cervical cancer developed by subcutaneous injection of 5 × 10^6 ^HeLa cells at the lower limbs of nude mice.** Arrows indicate the tumor lobes.

All animal procedures reported in the present paper were performed according to the NIH Animal Use and Care Guidelines (USA) and were approved by the INCan Ethics Committee. Female athymic Balb C nu/nu mice (6–8 weeks of age) used in this experiment were obtained from the Instituto Nacional de Nutrición (INNSZ), Mexico City, and were kept in a pathogen-free environment and fed *ad libitum*. The established, transplantable, HeLa human cervical cancer cell line was obtained from ATCC (Rockville, Maryland, USA) and was routinely maintained as a monolayer in Dubelcco's modified Eagles's medium (DMEM) with 10% fetal calf serum (FCS) (Gibco, BRL, Gaithersburg, MD, USA), and incubated in a 5% CO_2 _atmosphere and high humidity. Cells were harvested with 0.025% trypsin (Sigma-Aldrich Co., St. Louis, MO, USA) and 1 mM EDTA (Gibco, BRL). Animals were inoculated subcutaneously with 5 × 10^6 ^HeLa cells, suspended in DMEM without FCS, in both lower limbs. The limbs were selected as the site for tumor growth (Figure 2) to minimize irradiation to other body organs. After inoculation, tumors were measured, weekly, in two perpendicular diameters using a caliper and the tumor volume was determined by using the following relation:

V = π/6 × (large diameter × [short diameter]^2^) [17,18].

When tumor volume approximated 200 mm^3 ^the animals were pair-matched into treatments and control groups and the treatments were initiated.

### Conformal X-ray beam design and evaluation

**Figure 3:**
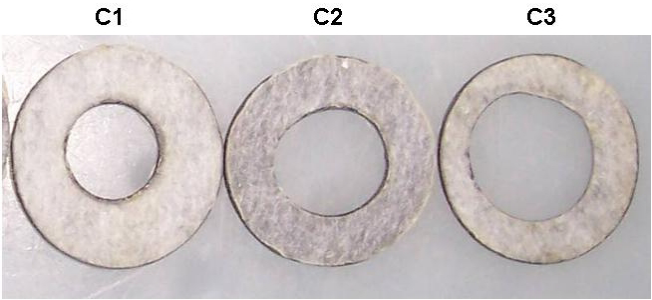
Lead collimators with different diameters (0.5, 1.0, 1.5 cm) and a thickness of 2 mm, used to restrict the X-ray beam onto the tumor lobes.

**Figure 4:**
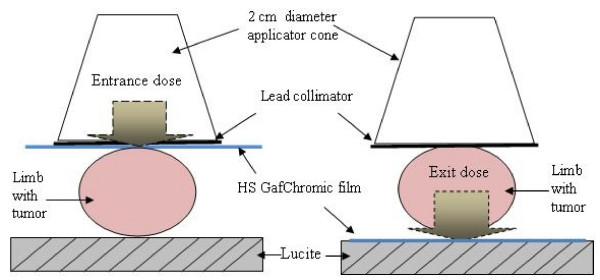
Experimental setup to evaluate the entrance and exit dose in the tumor lobe, using HS Gafchromic film.

To perform a conformal irradiation that will cover just the tumoral volume (typically 200 mm^3 ^grown in a mouse lower limb), lead collimators (2 mm thick) with 0.5 to 1.5 cm circular diameter apertures were manufactured (Figure 3) and mounted on the applicator cone to restrict the X-ray beam onto the tumor lobe. The thickness of the collimators was adequate to guarantee minimal penetration and dispersion of the X-ray beam outside of the collimator's aperture. The HVL for 60 keV photons in lead is 1.2 × 10^-3 ^mm [19]. X-ray penetration and conformal irradiation was evaluated by measuring dose at the surface (entrance dose) and behind the tumor lobe (exit dose) (Figure 4). HS GafChromic film was used to estimate the delivered dose.

### GafChromic irradiation and readout

Gafchromic films have been designed for use with gamma, x-ray and charged particles and can be used to measure and map radiation fields of a wide range of energies down to 5 keV [20,21]. When the film is exposed to ionizing radiation, a polymerization reaction in the film active layer is initiated resulting in the production of a blue-colored dye-polymer, and the amount of the color change is proportional to the dose absorbed by the active layer.

The GafChromic film (HS type) response as a function of dose (from 0 to 5 Gy) was measured using the same X-ray treatment unit; under the same conditions of tumor irradiations. This curve was used as a calibration to interpret the HS film response (*i.e*. the change in color).

Readout of HS GafChromic film followed a procedure previously described [22]. Briefly, film response was digitized 48 h after the end of irradiations with a Microtek ScanMarker 8700 scanner (Microtek Lab. Inc., USA) in transmission mode, using 36 bits RGB (12 bits per color), and saved as tagged image file format. The optical density range of the scanner was set to a maximum with all filters and image enhancement options turned off. The scanning resolution used was 300 dpi. Every film was scanned using an opaque frame to minimize light contributions from areas other than the film. Images were analyzed using the ImageJ (Version 1.36b) software (Wayne Rasband, NIH, USA). The film response, *R*, was quantified as:

$$R=Log_{10}\left(\frac{C_{ni}}{C_{i}}\right),$$

where C_ni _and C_i _are the measured color levels of the background (non irradiated) and irradiated films, respectively.

### Chemotherapy

Both cisplatin (Sigma-Aldrich Co., St Louis MO, USA) and gemcitabine (Eli Lilly and Co, USA) were reconstituted with sterile 0.9% saline in a laminar air-flow hood under sterile condition. Animals received four intraperitoneal cycles (1 cycle/week, on Mondays) of 2 mg/kg of cisplatin and/or 6.25 mg/kg of gemcitabine, concomitant with radiotherapy. Doses of both chemotherapeutic agents were adjusted from recommended doses used in the treatment of cervical carcinoma in humans (cisplatin: 40 mg/m^2^, gemcitabine: 125 mg/m^2^) [9] and the mass and body surface of mice and human adult females [23].

### Radiotherapy

Tumors were exposed to nominal 2.5 Gy entrance doses, 5 days/week, to complete 50 Gy. The dose and the schedule were selected because of their similarity to clinical patient treatment [9]. Tumor dose was estimated from average entrance and exit doses (measured with the HS film) taking into account the particular collimator used during the treatment. During irradiation, mice were anaesthetized with 1–3% isoflurane in 100% oxygen using an animal anesthesia inhalation unit (Bickford, Wales Center, NY). The X-ray beam was centered on the tumor lobe by using one of the different lead collimators, according with the tumor size at the moment of irradiation.

### Treatments groups

Animals which developed tumors in both extremities were randomized into three groups maintaining a similar tumor size distribution. Group A (n = 24 tumor lobes, i.e. 12 mice): treated with radiotherapy, cisplatin and gemcitabine as explained above; Group B (n = 26 tumor lobes): treated with radiotherapy and gemcitabine; Group C (n = 12 tumor lobes): untreated controls. Tumor volumes were measured as described, and a relative tumor volume (RTV) was determined using the relation: *RTV *= *V*_*i*_/*V*_0_, where *V*_*i *_is the weekly-measured tumor volume and *V*_0 _is the initial tumor volume (at the beginning of the treatment). The weight of each mouse was documented at each measurement to evaluate the toxicity of treatments.

### Clonogenic assay

Two weeks after the end of treatments, mice were sacrificed under isoflurane anesthesia and the tumors visibly present at the limbs were removed, washed in PBS and sectioned with surgical blade in sterile conditions. A mirror representative fragment was histologically analyzed with hematoxilin and eosin stain, to corroborate the presence of viable tissue, and the other fragment was used for clonogenic assay. The clonogenic *in vitro *assay was performed according the method described by Munshi A, *et al *[24] with modifications. The experiments were done by duplicate. Necrotic tissue was removed from the fragment, and isolated cells were obtained by mechanical fragmentation of the tumoral tissue and prepared into a single cell suspension by typsinization (PBS-EDTA 10 mM, pH 7.4, trypsin 0.2% (w/v)), 30 min 37°C with constant agitation. The cell number was obtained using blue trypan in a hemocytometer. A number of 5 × 10^4 ^cells were plated in Petri dishes containing high glucose-Dulbecco's modified Eagle's medium (DMEM, Gibco) supplemented with 10% bovine fetal serum (Gibco), 300 U/ml penicillin and 0.3 mg/ml streptomycin (Sigma). The cellular colonies were fixed with buffered formaldehyde (10.0% v/v), and stained with crystal violet (0.5% w/v). Proportion of clonogenic cells was determined by counting colonies 15 day later.

### Statistical analysis

Values are reported as mean ± SEM (standard error of the mean). Statistical analysis was performed using one-way analysis of variance (ANOVA) to compare the relative tumor volume and weight using SPSS Base 12.0 software (SPSS Inc, Chicago, IL). Differences were statistically compared using between-groups multiple comparisons. When necessary, comparisons among means were Bonferroni adjusted. A value of *p *<*0.05 *was defined as an acceptable probability for a significant difference between means. Statistical power of the ANOVA was calculated with a significant level of α = 0.05. A value lower than 0.5 was considered an insufficient power of the design for a medium size effect.

## Results

**Figure 5:**
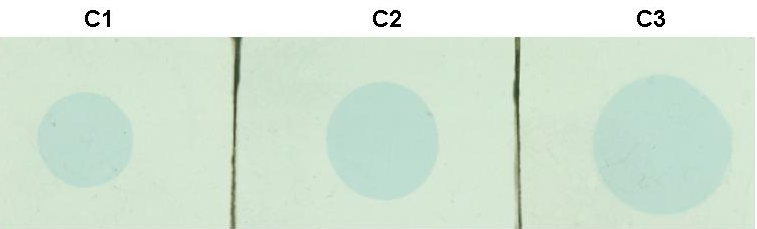
**Digitized images of HS Gafchromic film exposed to nominal 2.5 Gy, using collimators C1, C2 and C3 (see Figure**3).

Figure 5 shows photographs of irradiated HS GafChromic film used to estimate the uniformity of the delivered dose when the lead collimators were used. Analysis of the images shows that no measurable dose was delivered outside of the circular aperture of the collimators.

**Figure 6:**
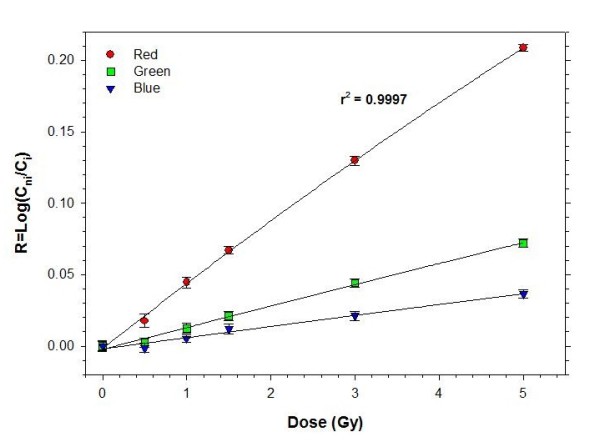
**Calibration curve (Response vs. Dose).** This curve shows the lineal response of the red, green and blue components (RGB mode) as function of the dose. Values are given as means ± SEM.

In Figure 6, the film calibration curve is presented. This figure shows the linear response of the red, green and blue components (RGB mode) as function of the dose in the measured interval. The red component was used in the analysis because of its higher sensitivity.

Table 1 show the measured entrance and exit doses when different collimators were used. Exposed HS films were scanned as described previously, and the image analysis was performed by drawing 3 to 5 regions of interest (ROI), of the same dimensions, in several positions around the central exposed area. No inhomogeneities in the response larger than 5% were observed across the films. An average value of the response in the ROIs was used to estimate the dose in each film by means of the calibration curve. In Table 1, results from all measured entrance doses are consistent with the nominal 2.5 Gy. No statistically significant differences among collimators are observed in the measured entrance or exit doses. The differences between the entrance and exit doses are consequence of the X-ray attenuation in the limb bone, tumoral and muscle tissues.

**Table 1:** Doses for 2.5 Gy nominal entrance dose measured with HS film and different collimators.

**Collimator**	**Diameter (cm)**	**Entrance dose (Gy)**	**Exit dose (Gy)**	**Entrance/Exit**
C1	0.5	2.47 ± 0.17	2.01 ± 0.17	1.23
C2	1	2.45 ± 0.15	1.98 ± 0.15	1.24
C3	1.5	2.50 ± 0.14	2.07 ± 0.17	1.21

**Figure 7:**
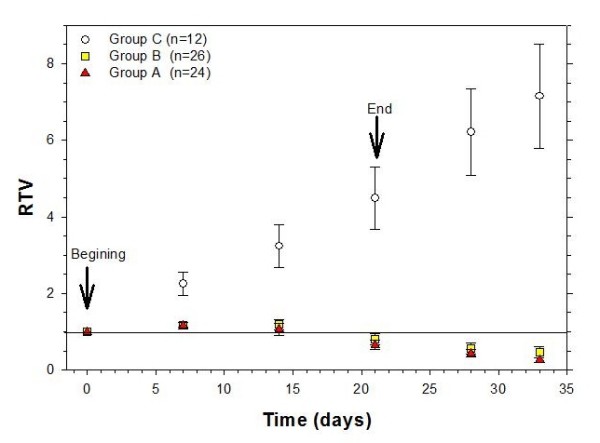
**Therapeutic response (Relative Tumor Volume vs. Time) for the control and the experimental groups.** Group A: cervical cancer xenografts treated with radiotherapy, cisplatin and gemcitabine as explained in the text; Group B: xenografts treated with radiotherapy and gemcitabine; Group C: untreated xenografts controls. Arrows indicate beginning and end of the treatments. Solid line signals RTV = 1, i.e. no change in tumor volume. Values are given as means ± SEM.

Figure 7 shows the relative tumor volume vs. time (i.e. therapeutic response) for the control and the experimental groups. The plot shows that the tumor lobe increases continuously in the control while practically disappears in the experimental groups. There was not difference in the therapeutic response between both experimental groups.

In Table 2, the number of colonies measured in the clonogenic assay is reported. The results show that no colonies were formed after the treatment with radiation and chemotherapy. These results was not due to necrosis or not viable cells, because the histological examination confirms the presence of macroscopic residual tumoral cells in almost the whole tissue samples employed in the clonogenic assay, although in some animals of groups A and B there was no evidence of neoplastic cells (8/16), in comparison with the control group (100%).

**Table 2:** Number of cell colonies measured in the clonogenic assay.

Group	Number of colonies
A	1 ± 1
B	0 ± 1
C	207 ± 5

Group A: cervical cancer xenografts treated with radiotherapy, cisplatin and gemcitabine; Group B: xenografts treated with radiotherapy and gemcitabine; Group C: untreated controls.

**Figure 8:**
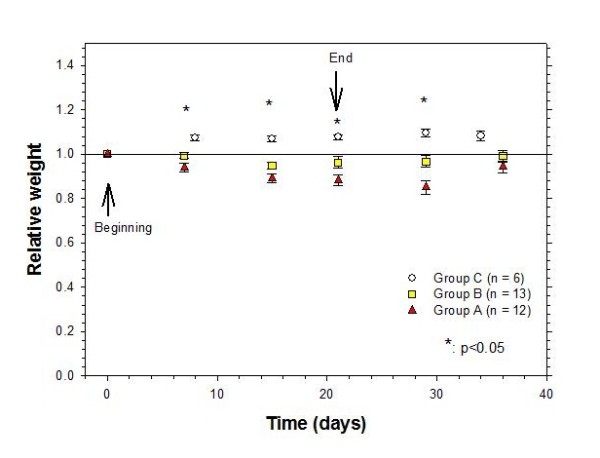
**Evaluation of the loss weight change along time.** Group A: cervical cancer xenografts treated with radiotherapy, cisplatin and gemcitabine; Group B: xenografts treated with radiotherapy and gemcitabine; Group C: untreated controls. Solid line signals a relative weight equal to 1, i.e. no change of weight. Values are given as means ± SEM.

Finally, Figure 8 shows the relative weight along the experiment time. The controls do not lose weight, they show an initial increase of around 8%, and afterwards the weight remained constant. In Groups A and B the loss of weight is evident after two weeks of treatment and becomes more pronounced for Group A, afterwards. Two weeks after the end of treatment, animals tend to recover their original weight. It is evident that the chemoradiotherapy treatment with cisplatin (Group A) results in a more significant loss of weight during the period of treatment, thus a higher toxicity of this treatment modality.

## Discussion

Previous published works of radiotherapy procedures in tumor models have reported the use of linacs, Co-60 and Cs-137 irradiators, or brachytherapy sources [5-8], which in the real experimental practice are difficult to operate with small animals. A main goal in this work has been to report that, while new dedicated radiotherapy systems for small animals are in the process of development and evaluation, clinical orthovoltage x-ray units can be used in radiotherapy experiments with tumor models.

The use of orthovoltage x-rays units has been more common in clinical veterinary therapy in pets (cats and dogs) [25] than in research procedures with tumor models in rodents, probably because the energies and range of the orthovoltage X-rays. Orthovoltage treatment units can produce X-ray beams with voltages in the range of 150 to 500 kV. At these energies, the maximum radiation dose occurs close to the skin, with 90% of that value occurring within a depth of about 2 cm [26], and the dose falling off rapidly into deeper tissues. For example, for X-rays having HVL = 0.5 cm of Cu (similar to this work), the depth for 50% of maximum dose is about 6 cm [16]. In the context of small animal tumor models, the small dimensions of the superficially grown tumor lobes (usually between 1 and 3 centimeters thick), imply that the delivered dose over the tumor volume will reach values from 80–95% up to maximum dose [26]. In the present experiment, the average thickness of the tumors lobes was around 1.5 to 2.0 cm. The results presented in Table 1 have shown that the differences between the entrance and exit dose were around 23%, due to attenuation of the X-ray beam in bone and soft tissues. This result suggests that dose inhomogeneities in the tumor reach ± 11.5% around the mean value which can be estimated as 11.5% lower than the nominal entrance dose. Within these assumptions, mean tumor doses in this study were approximately equal to 2.2 Gy, i.e. total dose of 44 Gy after 20 fractions.

It is well known that inhomogeneous radiation dose distributions in tumor may result in an incomplete elimination of tumor cells that may lead to an incomplete therapeutic response of the treatment, i.e. no reduction or elimination of the tumoral mass [27]. Same situation is observed with the inhomogeneous distribution of the chemotherapy agents in the tumor [28]. In the evaluation of the synergistic effects of the combination of chemoradiotherapy treatments in tumor models, one issue to evaluate is how homogeneities or inhomogeneities of dose distributions (radiative and chemotherapeutic) could affect the therapeutic response of the concomitant treatments; another issue is related with the doses used in both treatments. The results in this work have shown that a radiation dose of 2.2 Gy ± 11.5% daily results in an effective therapeutic response of the experimental groups in comparison with the control groups (*p *< 0.05, statistical power of 1.0 at α = 0.05). This effect could be consequence of the synergism between these treatment modalities, be related to the magnitude of the doses employed in either modality, or a combination of both. Future experiments are planned to evaluate and understand these results.

In this experiment we have used the simplest arrangement to show the advantages of the method employed with the orthovoltage X-ray unit. As part of the method evaluation, we have observed inhomogeneities around the mean dose. Obviously, an irradiation technique in two opposite directions should show further improvement in the dose uniformity, in consequence, it is recommendable the use of two opposite fields for similar works in cases when the quoted dose variation across the volume is unacceptable. The location of the tumor at the lower limbs will help in this kind of experiments.

When the chemotherapeutic treatments are compared, results have shown a reduction of the tumor volume as consequence of the X-ray irradiation with a radiosensitizer (Group B) or in combination with the chemotherapy agent (Group A). No difference in the therapeutic response was observed between these two groups (p > 0.05, statistical power < 0.5 at α = 0.05) in spite of the use of cisplatin in one of them. A more detailed study should be performed to analyze and understand this result.

The effectiveness of the chemoradiotherapy treatments, with the use of the orthovoltage system, was also validated with the clonogenic assay results, which show that treated tumoral cells lost their capability to form colonies and reproduce themselves. Finally, the results have also shown that the toxicity related with the concurrent chemoradiotherapy treatment, in terms of the weight loss, was well tolerated by the nude mice, allowing their recovery two weeks after the ending of the treatment. It is expected that the location of the tumor lobe at the mice lower limbs allows the reduction of radiation toxicity in normal tissues and organs.

## Conclusion

The results and procedures described in the present work have shown the usefulness of the orthovoltage X-ray treatment unit as a small-animal radiation system that allows the implementation of radiotherapy protocols in combination with other treatments in animal models.

## Competing interests

The authors declare that they have no competing interests.

## Authors' contributions

LAM and PGL planned the studies, made all coordination and were involved in the experimental procedures and performed results analysis; BIHP and MACM participated in the radiotherapy experimental design and chemoradiotherapy procedures; RJ participated in the tumor model design and chemotherapy procedures; EPC design and performed the clonogenic assay; JCV performed the histological analysis, MEB was involved in the dosimetry design, results analysis and manuscript revision. All authors read and approved the final manuscript.
